# Modern Medico-Psychology and Psychiatry

**Published:** 1894-09-22

**Authors:** T. S. Clouston

**Affiliations:** Lecturer on Mental Diseases, Edinburgh University, and Physician Superintendent Royal Asylum, Morningside


### Modern Medico-Psychology and Psychiatry.

By T. S. Clouston, M.D., Lecturer on Mental Diseases, Edinburgh University, and Physician Superintendent Royal Asylum, Morningside.

### XI.—CONDITIONS OF MENTAL EXALTATION AND EXCITEMENT.

Some "dour" and stolid men are only lively and agreeable when morbibly exalted. The whole condition strongly resembles the effects of moderate doses of alcohol on some brains. There is a mild auto-intoxication of the brain cortex. I knew a usually humdrum man, who, when passing through simple brain exaltation into an attack of acute mania, wrote a brilliant article for the most brilliant magazine of the time, which was published, the only such article he ever wrote in his life. Most of the cases tend towards a lower social level than the one in which they habitually live, during such attacks. Many of them being very susceptible to flattery, get into the hands of unscrupulous persons, who take advantage of their morbid state to rob them in some shape or form. One never sees human nature in so unattractive a shape as in the toady and sponger of the mildly maniacal or weak-minded patient. It seems as if there were a certain set of persons always on the watch for the uncontrolled and facile among their fellow creatures. They resemble bacteria, which are everywhere present, ready to take advantage of a suitable nidus whenever it appears.

This milder state of morbid mental elevation may not go on to something more decided, or it may advance to higher stages of elevation, to marked delusion, to incoherence of speech and ideas, or even to that complete dissolution of mind commonly called "delirious mania." When one sees it arrested and "recovered from," just as a cold or an inflammation is recovered from, it is possible for even an intelligent layman to realise that the organic vehicle of mind in the brain, has been undergoing a process of disease; as is the case also when the milder elevation has lasted for a month or two, and is seen to pass gradually into undoubted and unmistakable mania that no one could question—this, perhaps, killing the patient by exhaustion and want of sleep, as any acute disease may kill. In such cases its true nature from the beginning will, by any thoughtful person, be recognised. But it is often then too late; money, reputation, and position have been lost, and the chance of early and preventive treatment has been neglected.

Acute mania, by which term is denoted the higher degrees of morbid brain exaltation, is an extremely interesting disease to anyone who takes the trouble to study and analyse its symptoms, and to endeavour to understand their meaning. The human brain—that crown as yet of the whole process of evolution in nature—has by its hereditary potentialities, by its education, and by its environment, acquired capacities and qualities that are far more marvellous than the miracles of all the religions put together. Its power of correlation of all the facts and energies of nature, its stored up millions of sensations, its adaptation to what is without it, its organised ideas, its subjective re-actions to the objective world, its own creative and volitional capacity, make it the mystery of mysteries to the physiologist and phychologist. In acute mania through molecular changes in its cells, which we call pathological, but which are only an aggravation of the physiological, consciousness is altered and may be lost; volition is utterly paralysed; ideas become incoherent, sensations become changed, all the organised capacities of the organ become disorganised, the normal sequence of thoughts and feelings is lost, speech becomes useless to express meaning; the difference between the subjective and the objective, between the brain and its environment is no longer distinguishable; the difference between the real and the unreal no longer exists; thinking has ceased; the real ego has entirely changed with these mental symptoms. The body and organic functions of the brain are all changed. Sensation is altered in amount and quality, motion is tumultuous and uncontrolled, and nutrition impaired. The relationship of one function of the brain to every other function, constituting its solidarity, is lost. Its output of energy, mental and bodily, is wasteful and without purpose. Instead of its action being conservative to the whole of the rest of the organism, it is destructive, and often causes death. Its own functions have undergone dissolution, and it tends towards the dissolution of the life of the whole organism. "Duty," "motive," "instinct," "purpose," have no longer any meaning in the brain that is acutely maniacal.

Clinically the patient is ill; his temperature is up, in bad cases four or five degrees; his digestion, and his appetite are disordered; he is sleepless; he is losing weight, sometimes to the extent of three pounds a-day. There is not a secretion of the body but is chemically changed to some extent. The mental part of the brain is the highest and controlling centre that presides over every function and organ. These are all represented in the cortex side by side with mind. No wonder that an attack of acute mania, of "raving madness," is popularly regarded with terror. Yet it results from a pathological change in the brain cortex, a tissue which would not weigh altogether more than a few ounces, this causing all the symptoms mental and bodily which I have described. We can now demonstrate the changes in the brain cells in those cases which die of acute mania, just as we can demonstrate the changes in the kidney in Bright's Disease; but I shall reserve what I have to say about the pathology of mental disease for a special article.

